# The Links Between Ageism and the Age-Based Double Standard

**DOI:** 10.1093/geroni/igaa057.1885

**Published:** 2020-12-16

**Authors:** Changrui Li, Sarah Barber, Gene Brewer

**Affiliations:** 1 Georgia State University, Atlanta, Georgia, United States; 2 Arizona State University, Tempe, Arizona, United States

## Abstract

There is an age-based double standard in how we evaluate memory failures by younger and older adults. Whereas younger adults’ forgetfulness is attributed to lack of effort or attention, older adults’ forgetfulness is attributed to lack of ability. Our goal was to replicate this phenomenon, and evaluate its links to benevolent and hostile ageism. To do so, we used a vignette paradigm in which younger and older participants read about a target person (who was a younger or older woman) who left a store without paying for a ring (which varied in price). Results showed that participants were more likely to attribute this to poor memory abilities when the target was an older adult. They were also more lenient in their ascribed punishments for the older adult targets. In addition, reading about an older adult target’s mistake was associated with subsequently higher endorsement of benevolent, but not hostile, ageist attitudes.

